# Analysis of Injury Patterns and Outcomes of Blunt Thoracic Trauma in Elderly Patients

**DOI:** 10.7759/cureus.9974

**Published:** 2020-08-23

**Authors:** Nazish Sikander, Tanveer Ahmad, Khalil A Shaikh, Ambreen Abid, Misauq Mazcuri, Shagufta Nasreen

**Affiliations:** 1 Thoracic Surgery, Jinnah Postgraduate Medical Centre, Karachi, PAK

**Keywords:** blunt thoracic trauma, elderly, hemothorax, pneumothorax, outcome

## Abstract

Introduction

In the elderly population, trauma is a leading cause of utilization of healthcare, institutionalization, disability, and mortality. In this study, we will assess the injury patterns and the factors associated with the outcomes of blunt thoracic trauma in elderly individuals.

Methods

This prospective observational study was conducted in the Department of Thoracic Surgery, Jinnah Postgraduate Medical Center, Karachi, Pakistan, from September 2019 to April 2020. The study included patients of both genders aged 60 years and above with blunt thoracic trauma. Patients with concomitant neurological injuries and penetrating trauma to the chest were excluded.

Results

There were a total of 80 patients in this study; majority were males (n = 66 [82.5%]). The mean age was 70.18 ± 8.3 years. Road traffic accident was the most common mode of injury (n = 45 [56.3%]) followed by fall (n = 32 [40%]). Hemothorax and hemopneumothorax were the most common primary diagnosis. Rib fractures were encountered in 72 (90%) patients. Mortality rate was 21.3% (n = 17). Factors significantly related to mortality were age ≥ 80 years (p = 0.00), tension pneumothorax (p = 0.036), pre-existing cardiopulmonary disease (p = 0.032), blood loss ≥ 500 mL (p = 0.004), flail chest (p = 0.018), and chest trauma score ≥ 5 (p = 0.001). Mean hospital stay in our study was 5.3 ± 3.4 days. Factors lengthening hospital stay by more than five days included lung contusion (p = 0.02), more than two rib fractures (p = 0.004), hemopneumothorax (p = 0.026), pneumonia (p = 0.003), acute respiratory distress syndrome (p = 0.003), and flail chest (p = 0.013).

Conclusions

Elderly patients with blunt thoracic trauma have higher mortality. Proactive evaluation of injuries using the chest trauma score in the elderly population helps in recognizing patients at high risk of mortality and helps in the timely management to prevent adverse outcomes.

## Introduction

In the elderly population, trauma is a leading cause of utilization of healthcare, institutionalization, disability, and mortality [1]. At present, one-fourth (25%) of trauma-related hospital admissions are of the elderly [2]. By 2050, the percentage is expected to rise to 40% [3,4].

Blunt thoracic trauma (BTT) is frequently seen in the elderly and is associated with rib fractures and other injuries including hemothorax, pneumothorax, and pulmonary contusions [1]. Unintentional falls have also been reported to be a common cause of BTT, and increased age remains an independent predictor of mortality [5]. Reduced bone mineral density predisposes the elderly patients to increased rib fractures after thoracic trauma and may consequently result in a more prolonged and complicated clinical course [6]. Exposure to higher risk of traumatic events and significant co-morbidities result in higher injury sensitivity in geriatric patients [1]. Risk of intensive care unit (ICU) admission and intubation thus increase likewise [7]. Acute respiratory distress syndrome (ARDS) or worsening pulmonary sequel contributes to death in this geriatric population [7].

The current burden of elderly thoracic trauma is not known in Pakistan. According to the Pakistan National Emergency Department Surveillance (Pak-NEDS) study (November 2010 till March 2011), among all the fall-related cases presenting to the emergency departments, 3.1% were of age 65 years or more [8]. Keeping in view the scarcity of local scientific evidences amidst the rise of the elderly population in Pakistan, we initiated this study. Its aim was to assess the pattern of BTT in the elderly and factors predicting the outcome.

## Materials and methods

This prospective observational study was conducted in the Department Thoracic Surgery, Jinnah Postgraduate Medical Center, Karachi, Pakistan. The study duration was from September 2019 to April 2020. All patients were included after attaining informed consent.

**Figure 1:**
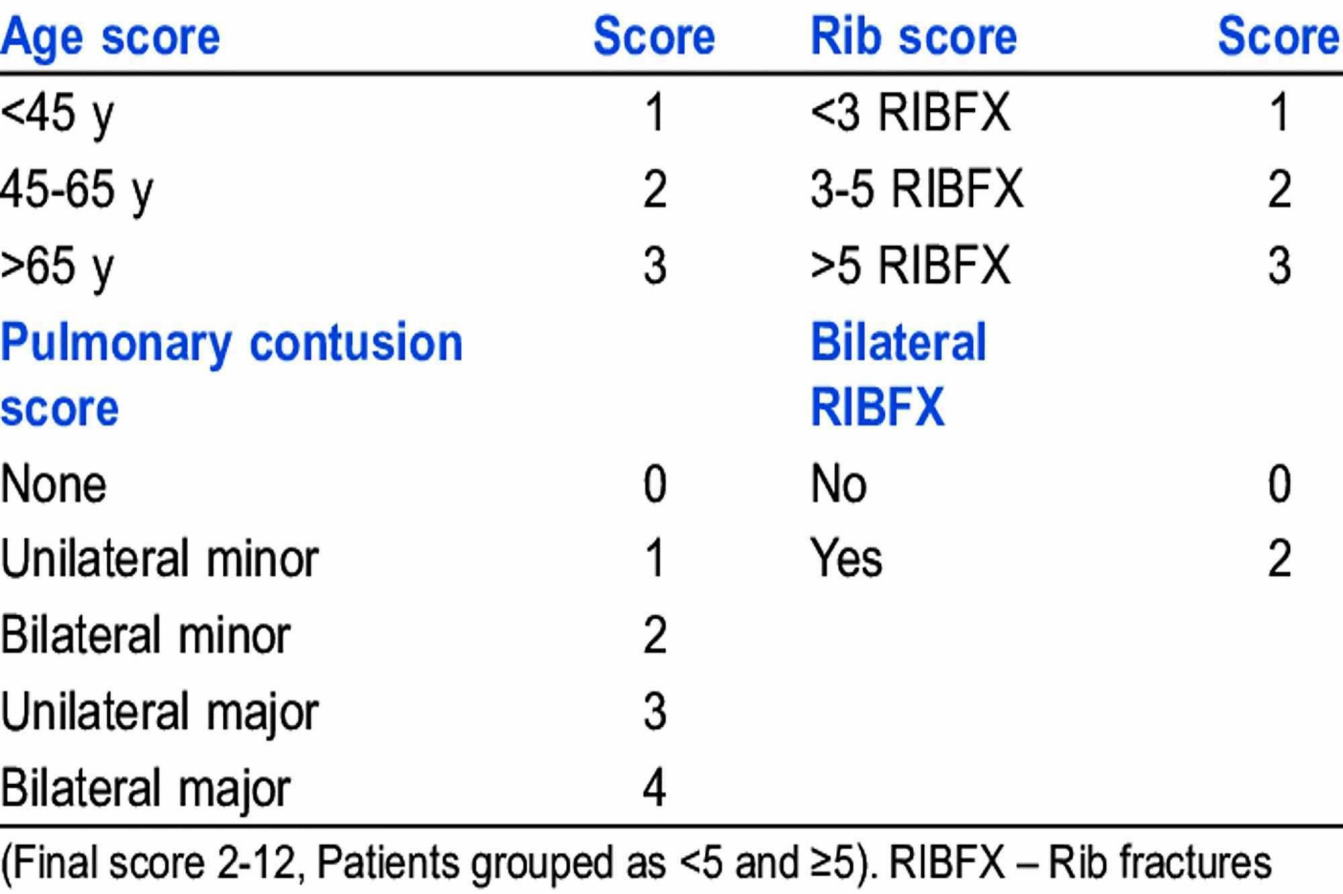
Calculation of the chest trauma score

The study included patients aged 60 years and above, of both genders, admitted with BTT. Patients with concomitant neurological injuries and penetrating trauma to the chest were excluded. Patients’ information was recorded in a semi-structured questionnaire, which included their sociodemographic characteristics, mode of trauma, primary and associated diagnoses, severity of trauma, and outcomes. Severity of trauma was assessed using the Chest Trauma Scoring (CTS) system, as shown in Figure 1 [9,10].

Outcome was assessed in terms of pneumonia, ARDS, need for invasive ventilation, and death. Data were analyzed using SPSS Version 23.0 (IBM Corp., Armonk, NY, USA). Mean and standard deviation was used to represent continuous variables. For categorical variables, frequency and percentages were calculated. Association among various study parameters were assessed using the chi-square test. A p-value of less than 0.05 indicates that the difference is significant enough to discard the null hypothesis.

## Results

A total of 80 patients were included in this study, of whom 66 (82.5%) were males and 14 (17.5%) were females. The mean age was 70.18 ± 8.3 years (range” 60-88 years). Road traffic accident (RTA) was the most common mode of injury (n = 45 [56.3%]) followed by fall (n = 32 [40%]). About 37 (46.3%) patients had cardiopulmonary conditions comprising ischemic heart diseases, hypertension, emphysematous lung, and chronic obstructive pulmonary disease. Hemothorax and hemopneumothorax were the primary diagnoses seen in 22 patients. Rib fractures were encountered in 72 (90%) patients, of which 65 (90.2%) had two or more rib fractures and 7 (9.7%) had less than two rib fractures. Patients were assessed using the CTS score. Of the 80 patients, 49 (61.3%) had a CTS score of <5 and 31 (38.8%) had a CTS score of ≥5. A significant association was noted between those who had two or more rib fractures and flail chest (p < 0.05) and the need for ventilatory support. Tube thoracostomy was performed in 66 (82.5%) patients, and 25 (31.3%) required emergency intubation and ventilation (Table 1).

**Table 1:** Characteristics of mode of trauma, primary diagnosis, and outcome of elderly patients with blunt thoracic trauma RTA, road traffic accident

Characteristics	Frequency (%)
Mode of Trauma	
RTA	45 (56.3%)
Fall	32 (40.0%)
Assault	2 (2.5%)
Others	1 (1.3%)
Primary Diagnosis	
Rib fracture	72 (90%)
Lung contusions	37 (46.3%)
Hemopneumothorax	22 (27.5%)
Hemothorax	22 (27.5%)
Pneumothorax	15 (81.3%)
Flail chest	10 (12.5%)
Tension pneumothorax	8 (10.0%)
Outcome	
Desaturation	25 (31.3%)
Intubation and ventilation	25 (31.3%)
Pneumonia	10 (12.5%)
Acute respiratory distress syndrome	10 (12.5%)
Mortality	17 (21.3%)

Mortality was seen in 17 (21.3%) patients. Factors related to mortality were age ≥ 80 years (p = 0.001), tension pneumothorax (p = 0.036), pre-existing cardiopulmonary disease (p = 0.032), blood loss ≥ 500 mL (p = 0.004), flail chest (p = 0.018), and CTS score ≥ 5 (p = 0.001). Rate of mortality and its association with trauma parameters are shown in Table 2.

**Table 2:** Relationship between mortality and parameters of trauma RTA, road traffic accident; CTS, chest trauma scoring

Characteristics		Mortality		p-Value
		No (n = 63 [78.7%])	Yes (n = 17 [21.3%])	
Age	60-69	37 (88.1%)	05 (11.9%)	0.001
	70-79	20 (83.3%)	04 (16.6%)	
	≥80	06 (42.8%)	08 (57.1%)	
Mode of injury	RTA	32 (71.1%)	13 (28.9%)	0.279
	Fall	28 (87.5%)	04 (12.5%)	
	Assault	02 (100.0%)	00	
	Others	01 (100.0%)	00	
Hemopneumothorax	No	46 (79.3%)	12 (20.7%)	0.84
	Yes	17 (77.2%)	05 (22.8%)	
Flail chest	No	58 (82.8%)	12 (17.1%)	0.018
	Yes	05 (50.0%)	05 (50.0%)	
Tension pneumothorax	No	59 (81.9%)	13 (18.1%)	0.036
	Yes	04 (50.0%)	04 (50.0%)	
Hemothorax	No	45 (77.5%)	13 (22.5%)	0.67
	Yes	18 (81.8%)	04 (18.2%)	
Pneumothorax	No	51 (78.46%)	14 (21.5%)	0.896
	Yes	12 (80.0%)	03 (20.0%)	
CTS score range	<5	45 (91.8%)	04 (8.2%)	0.001
	≥5	18 (58.1%)	13 (41.9%)	
Cardiopulmonary disease	No	37 (88.1%)	05 (11.9%)	0.032
	Yes	26 (68.4%)	12 (31.5%)	
Blood loss range	None	28 (82.4%)	06 (17.6%)	0.004
	<500 mL	01 (20.0%)	04 (80.0%)	
	≥500 mL	34 (82.9%)	7 (17.1%)	

CTS was used to assess mortality and morbidity, where 31 (38.8%) patients had a CTS score of ≥5. These patients showed statistically significant association with the need for ventilation (p = 0.001), pneumonia, ARDS (p = 0.014), and desaturation (p = 0.001) (Table 3).

**Table 3:** Relationship between chest trauma score and complications CTS, chest trauma scoring

Characteristics		CTS score range		p-Value
		<5 (n = 49 [61.2%])	≥5 (n = 31 [38.8%])	
Need for ventilator	No	47 (85.4%)	08 (14.5%)	0.000
	Yes	02 (8.0%)	23 (92.0%)	
Pneumonia	No	45 (68.1%)	21 (31.8%)	0.014
	Yes	04 (0.3%)	10 (71.4%)	
Acute respiratory distress syndrome	No	45 (68.1%)	21 (31.8%)	0.014
	Yes	04 (0.3%)	10 (71.4%)	
Desaturation	No	38 (74.5%)	13 (25.5%)	0.001
	Yes	11 (37.9%)	18 (62.1%)	

Mean hospital stay in our study was 5.3 ± 3.4 days. Factors lengthening hospital stay by more than five days were lung contusion (p = 0.02), more than two rib fractures (p = 0.004), hemopneumothorax (p = 0.026), pneumonia (p = 0.003), ARDS (p = 0.003), and flail chest (p = 0.013).

## Discussion

To the best of our knowledge, this is the first report on thoracic trauma in the elderly from Pakistan. According to the World Health Organization (WHO), around 1.3 million deaths worldwide were attributed to RTAs out of 55 million deaths in the year 2011 [11]. RTA is the ninth most common cause of disability-adjusted life years lost for all age and gender categories [12]. The distribution of deaths attributed to RTAs varies with age and sex, with males being the most vulnerable group, as also seen in our study [13]. RTA remains the most common cause of trauma in our study (56%). The second common cause of trauma in our study is reported to be fall-related injuries (40%). Other studies have also reported a high frequency of fall-related injuries in this geriatric population [5].

According to our study, the most common injuries following BTT are rib fractures, lung contusions, hemothorax, and hemopneumothorax. Similarly, in a study conducted at a tertiary care center on BTT in the elderly, rib fractures with associated hemothorax, pneumothorax, or hemopneumothorax were reported in as many as 72% of the patients [14]. In a three-year observational study that stratified patients according to their age, hemothorax and rib fractures were more common in the geriatric age group [15]. Due to the physiology of aging individuals, even minor trauma can have dangerous outcomes in these patients [16].

Flail chest is commonly encountered in BTT. It is seen to be associated with other injuries, most commonly pulmonary contusions [17]. In our study, 10 (12.5%) patients had flail chest, and all of them were associated with lung contusion. A retrospective analysis of injury patterns and clinical outcomes associated with flail chest indicated that the patients with sustained flail chest have significant morbidity and mortality [18]. Flail chest causes atelectasis, resulting in decreased lung volume due to inadequate and paradoxical chest movement. Alhadhrami et al. found out that epidural analgesia was enough to minimize the morbidity, mortality, and hospital stay in such patients. Unfortunately, we could not relate to it due to a lack of these facilities in the emergency department [19]. In our study, up to 50% mortality was seen in patients with flail chest (p = 0.018). Increased age (>65 years old) was found to be an independent predictor of mortality in these patients [20].

An important predictor of adverse outcome may include CTS score ≥ 5, which independently predicts mortality, pneumonia, and ARDS [9]. In our study, CTS was used to assess mortality and morbidity. Around 31 (38.8%) patients had CTS score ≥ 5 and showed significant association with complications such as pneumonia (p = 0.014), ARDS (p = 0.014), an increased need for ventilation (p = 0.00), and mortality (p = 0.001). Similarly, Harde et al. showed that CTS score > 5 is associated with poor outcomes and can be utilized for risk assessment in trauma patients to provide timely management [10].

Early ICU admissions should be considered for patients with BTT as it has been shown to improve the trauma outcome in these patients [21]. Failure of timely management with analgesia, physiotherapy, and respiratory support also increases the risk of complications including pneumonia, ARDS, and respiratory failure, resulting in the need for emergency intubation and ventilation [22]. Pulmonary contusions also increase the likelihood of adverse outcomes in these patients [23]. In our study, longer hospital stay was significantly associated with hemopneumothorax, amount of blood loss, flail chest, lung contusion, ARDS, pneumonia, and two or more rib fractures.

Geriatric care is entailing a challenge in the current health systems. With longevity, higher activity, and mobility, pre-existing medical conditions and aging have a profound influence on trauma care and outcome. The literature has established age to be an independent risk factor for mortality in trauma patients [5]. Harrington et al. studied the factors associated with improved survival in the elderly thoracic trauma. They reported that intubation, pre-existing heart failure, and admission to a high-volume trauma unit were the strongest predictors of mortality [22]. In our study, factors related to mortality were increasing age (p = 0.001), tension pneumothorax (p = 0.036), blood loss ≥ 500 mL (p = 0.004), flail chest (p = 0.018), and CTS score ≥ 5 (p = 0.001). In a retrospective analysis of thoracic trauma over 10 years, the incidence of thoracic injuries increased by 8% per year for the population and 14% per year for people aged ≥ 85 years [24].

Another factor predisposing to mortality in our study was preexisting cardiopulmonary disease (p = 0.032). This can be explained as elderly patients have decreased vital capacity, forced vital capacity, and forced expiratory volume, and therefore hypoxia and hypercarbia are poorly tolerated. Ambiguous response to hemorrhage and pain following trauma due to thickened myocardium, altered cardiac output, and desensitization to catecholamine’s masking heart rate may tamper the shock management [25].

In a recent systematic review, the paucity of data to establish prognostic factors for adverse outcomes of BTT in the elderly was highlighted [26]. This study has contributed to the existing literature of Pakistan. Geriatric care is still evolving in many developed countries, and in a low- to middle-income country like Pakistan, there remains a clear gap in specialized trauma care for the elderly individuals. This study lays the grounds for further efforts in this sector to enhance the level of geriatric healthcare and trauma, and in curbing mortality. It is recommended that trauma care providers and thoracic specialists investigate the age-related differences in thoracic trauma, which makes the elderly more vulnerable to adverse outcomes.

## Conclusions

BTT in the elderly is mostly caused by RTAs and unintentional falls. Injuries such as rib fractures, hemothorax, hemopneumothorax, and lung contusions were common. Patients with higher CTS scores were more prone to pneumonia and need for ventilation. Hemopneumothorax, flail chest, lung contusion, ARDS, pneumonia, and two or more rib fractures predicted a longer hospital stay. Age ≥ 80 years, tension pneumothorax, pre-existing cardiopulmonary disease, blood loss ≥ 500 mL, flail chest, and higher CTS score predicted mortality.

Elderly patients have higher morbidity and mortality following trauma. Proactive evaluation of injuries in the elderly population is compulsory, as it helps in the timely management of this age group and prevents adverse outcomes.
